# Tumor growth manifested in two-fifths of low-risk papillary thyroid microcarcinoma patients during active surveillance: data from a tertiary center in China

**DOI:** 10.3389/fendo.2024.1359621

**Published:** 2024-03-21

**Authors:** Kehao Le, Lei Jin, Fangfang Zhong, Xiaojuan Huang, Liang Zhou, Jiamin Zhou, Lei Xie

**Affiliations:** ^1^ Department of Head and Neck Surgery, the Affiliated Sir Run Run Shaw Hospital, Zhejiang University School of Medicine, Hangzhou, Zhejiang, China; ^2^ Department of Nuclear Medicine, the Affiliated Sir Run Run Shaw Hospital, Zhejiang University School of Medicine, Hangzhou, Zhejiang, China; ^3^ Department of Technology, Hangzhou KuaikuaiKangfu Technology Co., LTD, Hangzhou, Zhejiang, China

**Keywords:** papillary thyroid microcarcinoma, volume, active surveillance, tumor growth, tumor doubling rate

## Abstract

**Purpose:**

To assess tumor growth using tumor doubling rate (TDR) during active surveillance (AS) in China.

**Methods:**

Between January 2016 and June 2020, a total of 219 patients with low-risk papillary thyroid microcarcinoma (PTMC) (aged 23-75 years) were consecutively enrolled in the AS program.

**Results:**

Four sections of TDR, >0.5, 0.1~0.5, -0.1~0.1 and <-0.1, corresponded with four categories of tumor volume kinetics: rapid growth, slow growth, stable, and decreased size. We found that 10.5% of PTMCs exhibited rapid growth, 33.33% exhibited slow growth, 26.48% were stable, and 29.68% decreased in size. Tumor growth was associated with two factors: age and volume of PTMC at diagnosis. 85.72% of elderly patients (≥ 61 years old) had tumors that remained stable or even shrank and rapidly growing tumors were not found in them. When the volume was small (≤14.13 mm^3^), the proportion of rapid growth was high (41.67%), whereas when the volume was large (> 179.5 mm^3^), the proportion of non-growth was 68.75%.

**Conclusion:**

TDR may be a better metric for evaluating tumor growth in observational PTMCs. A certain proportion of PTMCs grow during the period of AS and tumor growth was associated with age and volume of PTMC at initial diagnosis. Therefore, how to block tumor growth during the AS period, especially for young patients and patients with early-stage PTMC (size ≤ 5 mm), will be a new challenge.

## Introduction

Papillary thyroid carcinoma (PTC) is the most common endocrine malignancy and it usually has an indolent biological nature. When the cancer measures ≤10 mm in diameter at its largest point, it is called a papillary thyroid microcarcinoma (PTMC). A rapid increase in the incidence of PTC has been reported in the past several decades in many countries. Approximately 50% of PTCs are PTMCs (1). However, a majority of PTMCs are occult and they are detected because of over screening in healthy populations. On this basis, in 1993, Dr. Miyauchi of Kuma Hospital in Japan proposed that low-risk PTMC patients could be followed with active surveillance (AS) rather than immediately undergoing surgery. The resulting study showed that the incidence rates of size enlargement, novel appearance of node metastasis, and progression to clinical disease in 1,235 PTMC patients were 8.0%, 3.8%, and 6.8% over a 10-year observation period, respectively (2). Therefore, presently AS is the first choice in managing low-risk PTMC in patients at Kuma Hospital (3, 4).

Following Kuma Hospital’s satisfactory clinical findings, more institutes began clinical trials around the world. In Korea, a retrospective cohort study, in which 192 PTMC patients were treated with AS, showed that some PTMCs could grow significantly during a relatively short period of observation and that changes in tumor volume were a more suitable metric for detecting tumor progression (5). American scholars did the first prospective AS study outside of Japan on 291 low-risk PTC (intrathyroidal tumors ≤1.5 cm) patients. They found that the rate of tumor growth during AS was low and AS could be successfully implemented outside of the Japan (6). Additionally, scholars from the University of Pisa presented a study in which only 3% of PTMC patients (out of 93) demonstrated disease progression. This study had a median 19-month AS. This shows that within an Italian medical context active surveillance appears to be a feasible and safe alternative to immediate surgery (7). However, up to now, few similar clinical PTMC with AS studies have been published in China.

This study is a prospective one on Chinese PTMC patients with AS. We analyzed the data from a tertiary A-level hospital and assessed the tumor growth by tumor doubling rate (TDR) during AS in China.

## Patients and methods

Between January 2016 and June 2020, a total of 219 patients with low-risk PTMC (aged 23-75 years) were enrolled in the AS program, which was approved by the Hospital Ethics Committee of Sir Run Run Shaw Hospital as part of the Medical School of Zhejiang University, P. R. China. The deadline for follow-up was June 2021 and each patient was checked by ultrasound at least twice. Low-risk PTMC patients with AS were defined and treated as follows:

1 tumor size < 1cm in maximal dimension at time of diagnosis.

2 thyroid nodules diagnosed as Bethesda category IV, or V with suspicious ultrasonographic features (Chinese Guidelines for Ultrasound Malignancy Risk Stratification of Thyroid Nodules [C-TIRADS] ≥ 4c) and BRAF V600E mutation.

3 no imaging or cytological evidence of invasion of local structures, regional lymph nodes or distant metastasis.

4 cytological interpretations by senior thyroid cytologists at Sir Run Run Shaw Hospital.

5 ultrasound examination every 6 or 12 months by senior thyroid sonographers at Sir Run Run Shaw Hospital.

6 all clinical data legally collected and managed by 17 CARE, a digital health evaluation network platform.

7 informed-consent forms signed by all the patients and each PTMC patient was given a 3 or 6-month hesitation period before he or she joined the clinical trial.

8 only the largest nodule (found in the most recent inspection) was calculated when the PTMC had multiple lesions.

The tumor was measured in three dimensions by advanced ultrasound. Meanwhile, the ultrasonographic features of the tumor were analyzed. Tumor volume was calculated using V= π/6 × length × width × depth. A Doubling Time & Progression Calculator (available from: http://www.kuma-h.or.jp/english/) was used to calculate the volume change and two definitions of doubling time and rate were used (8). If the maximum size of the primary tumor increased by 3 mm or more from the initial size, or reached more than 1cm, or there was evidence of lymph node or distant metastasis, surgical treatment was recommended.

### Statistical analysis

A Student’s t test or the Mann-Whitney test was used for statistical analyses, as appropriate. χ^2^ tests and Fisher’s exact probability tests were used to compare frequencies. p < 0.05 was regarded as a significant difference.

## Results

Between January 2016 and May 2020, 219 patients (48 men and 171 women) (aged 23 – 75 years) with low-risk PTMC were enrolled in our hospital’s AS program. As of June 2021, they had been followed for at least 1 year and the mean follow-up time was 34.7 months. No patients developed extra thyroid invasion, lymph node metastasis or distant metastasis during follow-up period.

The growth of the tumor is regarded as exponential and tumor doubling time (TDT) is suggested to describe the tumor growth rate (9). To overcome some limitations of TDT, 1/TDT value is used and called TDR because it indicates the number of doublings or halving that occur per unit of time (8). When TDR is 0.5, TDT is 1/0.5, which means that the doubling time of the tumor volume is 2 years. When TDR is negative, it means that the tumor is shrinking.

**Figure 1:**
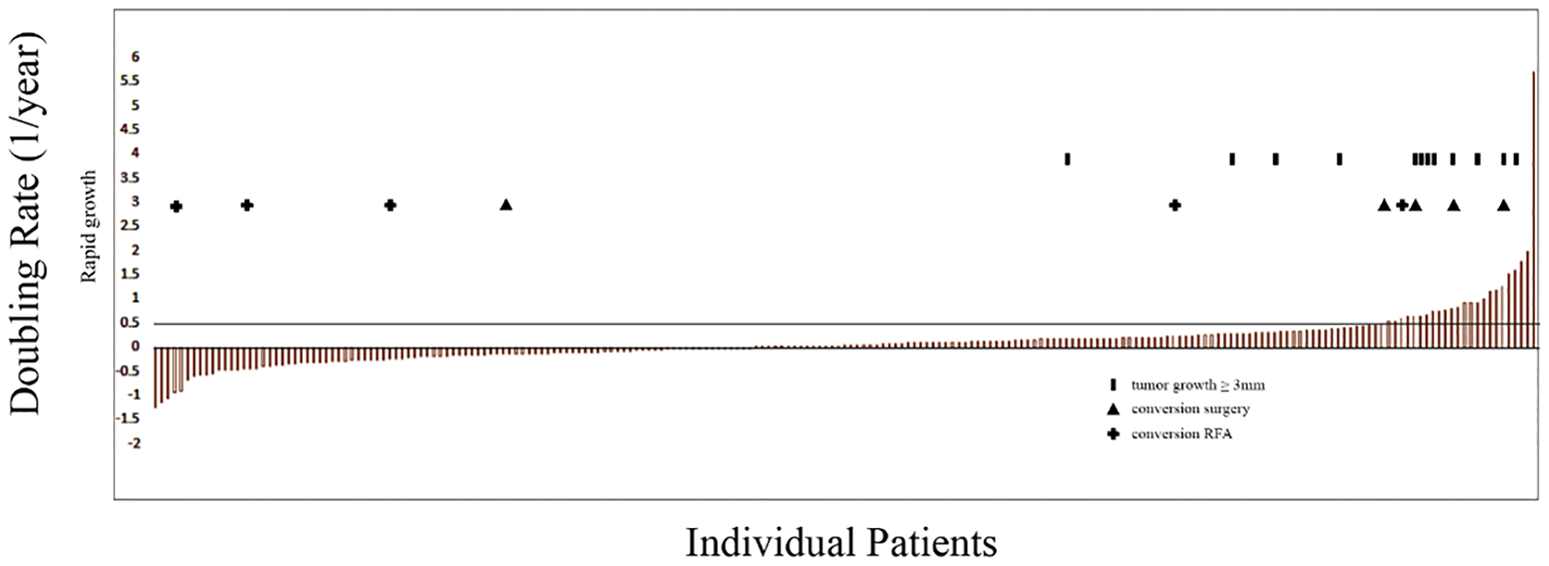
Doubling rates indicating rapid tumor growth (>0.5) during active surveillance (AS) for each patient; columns represent patients retained in AS; solid columns indicate patients whose tumors increased ≥3 mm in size; + indicate patients who underwent conversion from AS to radio-frequency ablation; triangles indicate patients who underwent conversion from AS to thyroid resection.

According to Kuma Hospital’ s standard, the cutoffs for doubling rates of 0.5, 0.1 and -0.1 per year were used. Four sections of doubling rates were >0.5, 0.1~0.5, -0.1~0.1 and <-0.1, corresponding to four categories of the tumor volume kinetics: rapid growth, slow growth, stable, and decrease in size. Analyzing our data, we found that 10.5% of PTMCs exhibited rapid growth, 33.33% exhibited slow growth, 26.48% were stable, and 29.68% exhibited decrease in tumor size (**Table 1**). There were 12 patients whose tumors increased ≥3 mm in size, including 8 in 23 patients with rapid growth and 4 in 73 patients with slow growth (**Figure 1**).

**Table 1:** Change in doubling rate of papillary microcarcinoma during follow-up.

Follow-up onset(yr)	No. Of PMC	Change in doubling rate of the tumor			
		A≥0.5	0.1≤A<0.5	-0.1≤A<0.1	A<-0.1
2016	10	1	3	6	0
2017	38	6	18	4	10
2018	66	4	23	18	21
2019	84	7	25	23	29
2020	21	5	4	7	5
Total	219	23(10.50%)	73(33.33%)	58(26.48%)	65(29.68%)

Duration of follow-up and outcomes.

The patients were also divided into three groups: rapid growth, slow growth, and no growth, corresponding to the three sections of doubling rates: >0.5, 0.1~0.5 and <0.1. Then, the clinical feature difference was further analyzed among the three groups, and we found tumor growth was significantly associated with two factors: age and volume of PTMC at diagnosis (**Table 2**). The results were further described as follows:

**Table 2:** Relationship between outcomes in doubling rate of observation and clinical factors and for papillary microcarcinoma.

Clinical characteristics	Total	Tumor doubling rate decreased or increased less than 0.1 (n=123)	Tumor doubling rate increased more than 0.1but less than 0.5 (n=73)	Tumor doubling rate increased more than 0.5 (n=23)	p value
Age at diagnosis (yr)	42.56 ± 11.64	44.50 ± 12.60	40.60 ± 9.80	38.20 ± 8.50	**<0.05**
Male/female ratio	48/171	30/93	13/60	5/18	NS
Duration of follow-up (month)	34.7 ± 18.2	33.2 ± 17.3	39.2 ± 18.2	31.0 ± 20.4	NS
Duration of follow-up (yr)	2.9 ± 1.5	2.8 ± 1.4	3.3 ± 1.5	2.6 ± 1.7	NS
Hashimoto’s thyroiditis (absent/present)	89/35	47/23	33/7	9/5	NS
Maximum diameter of PMC at diagnosis (mm)	5.6 ± 1.6	6.0 ± 2.2	5.4 ± 2.2	4.7 ± 1.9	NS
Volume of PMC at diagnosis(mm^3^)	63.17 ± 45.6	103.1 ± 74.4	118.2 ± 131.6	32.4 ± 23.5	**<0.05**
Multiple lesions (absent/present)	50/169	29/94	17/56	4/19	NS

The bold values indicate the significant difference. NS, non-significant.

1 Age

**Figure 2:**
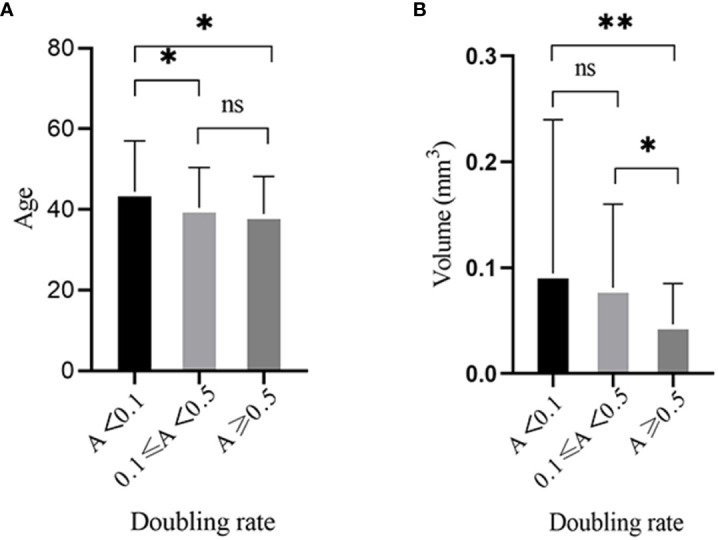
Tumor growth was significantly associated with three factors: age and volume of PTMC at diagnosis. **(A)** There was significantly difference between the no growth group and the other two groups and there was no difference between slow and rapid growth groups in regards to age. **(B)** The volume of PTMC at diagnosis was significantly different between the rapid growth group and the other two groups, and no difference was found between the slow and no growth groups. *p<0.05, **p<0.01, ns, no significant difference.

The age showed significantly different between no growth group and the other two groups, and there was no difference between slow and rapid growth groups (**Figure 2A**). We further divided the patients into three age groups: ≤40 (youth), 40~60 (middle aged) and ≥61 (elderly) and analyzed the tumor growth status in them. We found that more than 85% of tumors didn’t show any enlargement and no tumor showed rapid growth in the elderly group. In addition, the proportion of slow growing tumors decreased with the increase of age (**Table 3**).

**Table 3:** Change in doubling rate of papillary microcarcinoma (PMC) during follow-up according to age at presentation.

Age,(years)	No. Of patients	Change in doubling rate of the tumor		
		A≥0.5	0.1≤A<0.5	A<0.1
≤40	116	14(12.07%)	45(38.79%)	57(49.14%)
41-60	82	9(10.98%)	25(30.49%)	48(58.53%)
≥61	21	0(0%)	3(14.29%)	18(85.72%)
Total	219	23(10.50%)	73(33.33%)	123(56.16%)

Duration of follow-up and outcomes.

2 Volume of PTMC at Diagnosis

The volume of PTMC at diagnosis was significantly different between the rapid growth group and the other two groups, and no difference was found between the slow and no growth groups (**Figure 2B**). Assuming the length, width, and depth of the tumor were the same, when the length was 3, 5, and 7mm, the volume was 14.13, 65.42, and 179.5mm^3^, respectively. Then we further analyzed the tumor growth status within different volume ranges. We found that the incidence of rapid growth in the PTMCs with ≤14.13 mm^3^ was the highest and up to 41.67%; whereas most of the tumors with >179.5 mm^3^ (68.75%) showed no growth (**Table 4**).

**Table 4:** Change in doubling rate of papillary microcarcinoma (PMC) during follow-up according to volume at presentation.

Volume(mm^3^)	No. Of patients	Change in doubling rate of the tumor		
		A≥0.5	0.1≤A<0.5	A<0.1
≤14.13	12	5(41.67%)	4(33.33%)	3(25.00%)
14.13-65.42	108	10(9.26%)	38(35.19%)	60(55.56%)
65.42-179.5	83	8(9.64%)	26(31.33%)	49(59.04%)
>179.5	16	0(0)	5(31.25%)	11(68.75%)
Total	219	23(10.50%)	73(33.33%)	123(56.16%)

Duration of follow-up and outcomes.

In January, 2021 ten of the 219 patients subsequently underwent conversion to thyroid resection or radio-frequency ablation (RFA). The long diameters at final examination were less than 5mm in all 5 cases of RFA, and there were 3 cases with TDR <-0.1 and 2 cases with >0.2. However, in 4 of 5 cases with surgery the long diameters at final examination were about 1cm and the TDRs were ≥0.5 (**Supplementary Table 1**) and another case was converted to operation due to the tumor being near the recurrent laryngeal nerve. In addition, a previous study showed both consolidation of calcification and loss of vascularity were significant indicators for non-progressive disease (10). So, we also analyzed the ultrasonographic findings of PTMC among the three groups and no difference was found. (**Supplementary Table 2**).

## Discussion

Referring to Kuma Hospital’s experience, we utilized TDR to describe the tumor growth status and found that a certain proportion of tumors were increasing in size during a follow-up period and the proportion was not low: 10.5% showed rapid growth and 33% showed slow growth. With further analysis, we found tumor growth was associated with two factors: age and volume of PTMC at diagnosis. In previous studies it has been demonstrated that patient age is significantly related to the progression of PTMC: PTMC in young patients may have more progression than in older patients. In our study we got a similar result, which showed that 85% of elderly patients (≥ 61 years old) had tumors that remained stable or even shrank and rapid tumor growth was not found in them. In their study on sonographically suspicious thyroid nodules (1cm or smaller) with AS, Hu Y.L. et al. found that nodules with smaller volumes at diagnosis were more likely to increase in volume later (11). In the previous study on the natural history of PTMC, the estimated doubling rate before presentation converted from a hypothetical TDT were significantly greater than the doubling rates calculated during AS, so Miyauchi A. et al. inferred that there was a rapid growth period before PTMC clinical presentation (8). Our result demonstrated their inference, namely, the proportion of rapid growth was the highest when the volume of tumor at diagnosis was less than 14.13mm^3^, which could be regarded as PTMC’s pre-clinical presentation.

LB Woolner and his colleagues from the Mayo Clinic were the first to coin the term occult papillary carcinoma, indicating PTC≤ 1.5 cm in size in 1960 (12). Their study showed the good prognosis of occult papillary carcinoma through a 30-year follow-up of 140 cases. In 1989, the WHO introduced the term papillary microcarcinoma to replace occult papillary carcinoma and it was defined as PTC ≤1 cm in diameter. PTMC was regarded as an important variant of PTC because of low malignancy and exceptionally rare distant metastasis, 6%~35% frequency as incidental findings in autopsy studies and increasing frequency in life by modern methods of investigation. After that, most of the studies on PTMC showed excellent prognoses in many different centers (2, 13). Therefore, we must ask a question: Is immediate surgery the only right way for all PTMC patients? Presently we must be confronted with such a dilemma: whether thyroid cancer screening is worthwhile or is thyroid microcarcinoma over-diagnosed and over-treated? This dilemma profoundly reflects a contradiction between many PTMCs found through ordinary health checks and their improper treatment. Immediate thyroidectomy is considered the only “correct” measure by many clinics. Meanwhile, we must admit that while many low-risk PTMCs are “mined” through health screenings, some high-risk PTMCs and advanced thyroid cancers are also found. How can we overcome this dilemma? We seriously put forward that preservation of normal thyroid function should be regarded as a new philosophy of low-risk PTMC treatment, rather than the traditional radical resection.

**Figure 3:**
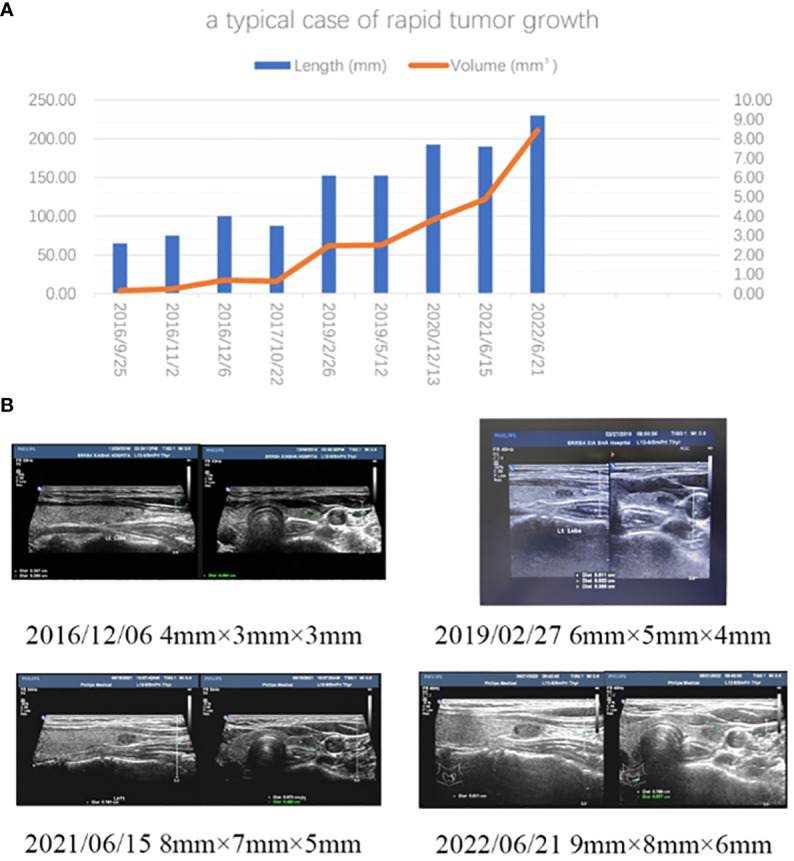
**(A)** A typical case of rapid tumor growth in observational PTMCs. The patient was a 30-year-old woman. A small nodule (4mm×3mm×3mm) in the inferior pole of her left thyroid was found by ultrasound during a health examination in 2016. Bethesda VI was diagnosed by FNA in 2016. During AS period, it gradually grew to the size of 9mm×8mm×6mm. It could be foreseen that, according to her TDR (0.92), she would have to be treated with thyroidectomy sooner or later in her life due to the tumor progression during the observation period. If this nodule were ablated by ultrasound-guided hyperthermia at the initial diagnosis, she would have avoided thyroid resection and preserved her thyroid function. **(B)** Ultrasound images taken at different times.

Active surveillance of low-risk PTMC reflects this change in treatment methodology to a certain degree, and the results of a 10-year follow-up (2) and 30-year experience (14) are encouraging. However, we still confront two problems in our clinical practice of AS. One is that a certain proportion of PTMC patients with AS will be treated with surgery in their lifetime because of clinical progression, which is based on data analysis and prediction (2). This proportion is not low, 20% to 50% of patients who presented PTMC between 25 and 45 years of age. We found more than 40% of PTMCs increased in size in this study. The other is that the tumor size is significantly associated with the progression or invasive characteristics of PTMC. As detailed in our previous studies (15), it was found that the incidence of LN metastasis was doubled when the tumor grew from 5mm to 10mm. Analyzing 997 PTMC cases, Zheng XQ and his colleagues found that tumor size (>5mm) was an independent risk factor associated with LN metastasis (16). As a result, we believe that if PTMC patients can be treated by local ablation at the time of initial discovery or at a very early stage (small tumor size, such as ≤5mm), they are very likely to avoid thyroidectomy and thyroxine supplementation due to clinical progression during AS in their lifetime (**Figure 3**). This is the new value of ultrasound-guided thermal ablation (UGTA) in the new treatment strategy for PTMC. The new strategy should not simply be destroying the tumor but, should rather be blocking tumor development to avoid surgery in a patient’s lifetime. We completely agree with the scholars from Kuma Hospital that non-surgical treatment should be considered first for low-risk PTMCs and AS is a reliable and efficient management strategy. Moreover, UGTA might be a good option too, especially for younger PTMC patients and/or smaller tumor lesions (≤5 mm in size). Therefore, based on this logical thinking, 5 cases of small PTMCs (**Supplementary Table 1**) were treated with RFA in the later stage of this trial.

Our data was collected and analyzed at the end of 2021, and the article was written during the whole year of 2022. We explored various indicators, including long diameter (>3mm), >50% volume growth, and TDR, to evaluate the tumor growth status. Ultimately, we found that TDR might be the most suitable indicator to reflect PTMC’s tumor volume change and trend due to its three-dimensional changes over time. We believe that its description of tumor growth may offer a more comprehensive and realistic perspective than other indicators. Do we really need this sensitive indicator, or what is the value of the usage of it to describe the tumor growth? 10 years ago, the options for treating PTMC were limited to AS or surgery, with AS often equated to “delayed surgery” (8). Consequently, using more sensitive indicators to assess tumor growth was not deemed beneficial at that time. We find that the diameter of PTC that can be observed is constantly extending: 1 cm for ATA (17), 1.2 cm for Kuma Hospital (2), 1.5 cm for Dr. Tuttle RM (18), and even 2 cm in some literature (19). However, with the emergency of UGTA, such as RFA, as a third method for PTMC treatment, the landscape has changed. There are many research reports about using PTMC treating with RFA, and the conclusion is that thermal ablation is an effective and safe option for the management of low-risk PTMC (20). Despite reservations from some scholars, especially thyroid surgeons (21), regarding RFA in PTMC treatment, RFA’s role in PTMC treatment should not be underestimated based on the PTMC’s biological behavior, the thyroid gland’s anatomical location, AS clinical trials results, and the RFA mechanism. When a malignant lesion shows a tendency to increase in size, choosing active treatment like local thermal ablation becomes a viable option over a passive “wait and see” approach.

**Figure 4:**
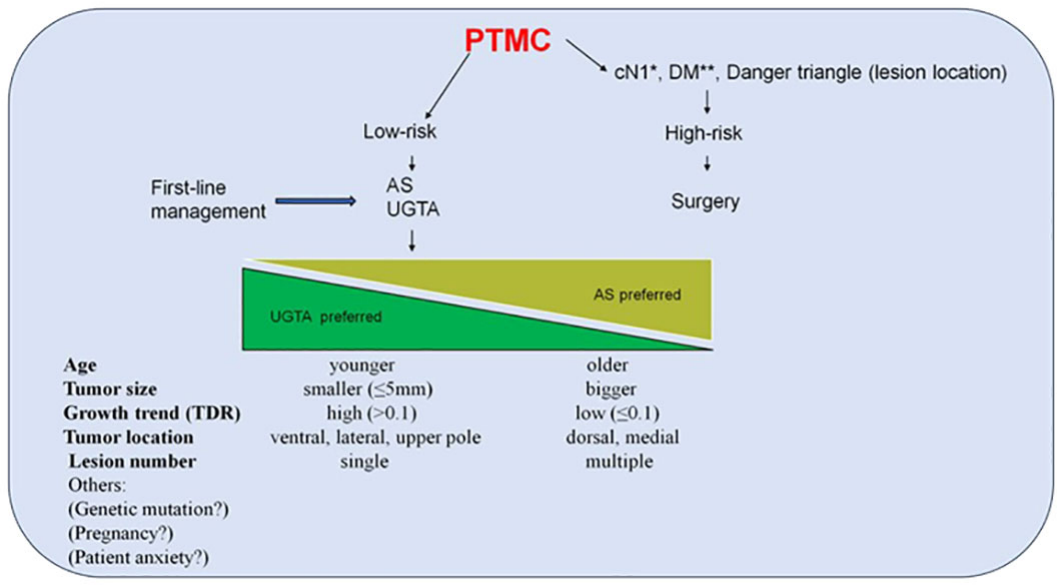
The preliminary framework of PTMC treatment stratification. This framework is a preliminary idea to integrate these three treatments, including surgery, AS and UGTA. UGTA should be preferred over AS if the patient is young, the lesion is small, TDR is high and the location is appropriate. Compared to tumors located on the dorsal or medial side, PTMC located on the ventral or lateral side is more suitable for RFA because it is far from the recurrent laryngeal nerve. For PTC, upper pole location is a risk factor for lateral neck lymph node metastasis, so the PTMC in the upper pole in its early stage should be treated with RFA instead of AS to avoid the possibility of this kind of metastasis. Compared to many small lesions in the thyroid gland, a single lesion is more suitable for RFA, rather than AS. Finally, other factors should be also considered to choose AS or RFA, such as genetic mutations, pregnancy and patient anxiety. *Neck lymph node metastasis confirmed clinically. **Distant metastasis. AS Active surveillance; UGTA, Ultrasound-guided thermal ablation; RFA, Radiofrequency ablation.

Currently, thyroid lobectomy, AS, and UGTA are recognized as the three methods for treating PTMC. Dr. Tuttle RM and his colleagues have introduced a new definition, referring to it as the minimalistic management option (22). They suggest establishing a clinical framework that integrates three characteristics of imaging, patient, and medical team characteristics to classify a patient as ideal, appropriate, or inappropriate candidates for these three treatments. However, their idea is rooted in the traditional treatment concept of radical tumor resection, and these three treatment methods are viewed as polar opposites. As we mentioned earlier, once the treatment philosophy is transformed into protecting thyroid function, these methods can truly be integrated together to achieve stratified PTMC treatment. In summary, non-surgical treatment, including AS and RFA, should be the first line of treatment, while surgical treatment should be reserved for high-risk PTMC cases, such as distant metastasis, lymph node metastasis, and special tumor locations. The choice between AS and RFA should consider factors such as patient age, tumor size, growth trend, location, patient preferences and so on. RFA should be preferred over AS in cases of young patients, small lesions, high TDR, and appropriate locations. (**Figure 4**)

Further points about this study’s results worth mentioning: First, the evaluations of LN metastasis in the neck by our senior sonographer were very thorough for each PTMC patient, especially for suspicious LNs. FNA with Tg washout was performed. The other reason was that the follow-up time was relatively short. Therefore, no cervical LN metastasis was found during AS in this cohort. Second, each PTMC patient was given a 3 or 6-month hesitation period before he or she joined this clinical trial and they had enough time to understand the pros and cons of AS or thyroidectomy. Therefore, after enrollment, few patients had to undergo surgery due to anxiety about the disease. Third, considering ultrasound shadowing from intra-tumor macrocalcification, tumor depth might be not reliable to be measured. So, tumor volume was calculated using V = π/6× length × width ×width in Kuma Hospital (8). However, macrocalcifications were found in less than 20% PTMCs in our data (**Supplementary Table 2**), so the length, width and depth of the tumor were still measured and calculated, in order to accurately reflect the three-dimensional of the lesion. Fourth, about 30% PTMCs occurred tumor regression in our data. This phenomenon was also found in Dr. Miyauchi (8) and Dr. Tuttle’s clinical trials (18). A scholar speculated that fine needle aspiration might cause some infarction in the lesion, or some bleeding resulting in becoming bigger in initial tumor size. Whereas another scholar thought that the tumor shrinkage might be a natural process (8). We felt that these might all exist. This issue will be worthy of further study. Fifth, our result showed that 33.33% of PTMC cases exhibited slow growth, which was different from some reports from other centers. The factors that led to this discrepancy were complicated, including different formulas for calculating volume (8), different indicators (2, 4) for assessing tumor growth, and so on. Dr. Tuttle RM and his colleagues had described six most common tumor volume kinetic patterns of PTC during the period of AS (23). They found that the incidence of tumor growth, including Pattern II~V, was only 16.6%. However, it was noteworthy that compared to our data, their age was usually older and the tumor was usually larger. Age and initial tumor size were the most important factors leading to tumor growth, so we believed this might be the reason for this difference. Sixth, compared with current research on AS in PTMC, in our clinical trial, the number of patients was relatively small and the follow-up time was relatively short, and a selective bias might exist in this study due to inconsecutive enrollment. In order to obtain more convincing evidence, we will continue to develop this study in multiple medical centers in China.

The clinical trial of PTMC with AS has evolved globally for the past 30 years. 30 years ago, Dr. Miyauchi A. proposed two hypotheses to characterize the natural history of PTMC (24): one posits that PTMC is an early stage of clinical cancer that will eventually prove fatal, while the other suggests that PTMC remains small and is therefore harmless. Our studies, in conjunction with those conducted at Kuma Hospital, focus on utilizing TDR to assess tumor growth status. We contend that both hypotheses align with the clinical manifestations of PTMC, each accounting for a certain proportion (8). Currently, the latter hypothesis receives more attention, while the former is often overlooked. In fact, the proportion of the former is not insignificant. For this reason, promoting AS in routine clinical practice poses challenges for doctors. Cari M. Kitahara and his colleagues, analyzing data from SEER-18 covering 28% of the U.S. population, found that non-surgical management was employed in less than 1% of cases of PTCs <1cm during 2000-2018 (25). In Kuma Hospital’s latest study, 3222 PTMC patients participating in the AS clinical trial, only about 62% remained on AS treatment (14). Therefore, we believe that how to avoid thyroid resection due to the clinical progression of observational PTMC during the life cycle will become a very important issue that we need to consider, especially when following a new treatment philosophy and method.

## Data availability statement

The raw data supporting the conclusions of this article will be made available by the authors, without undue reservation.

## Ethics statement

The studies involving humans were approved by Hospital Ethics Committee of Sir Run Run Shaw Hospital as part of the Medical School of Zhejiang University, China. The studies were conducted in accordance with the local legislation and institutional requirements. The participants provided their written informed consent to participate in this study. Written informed consent was obtained from the individual(s) for the publication of any potentially identifiable images or data included in this article.

## Author contributions

KL: Data curation, Formal analysis, Funding acquisition, Methodology, Resources, Software, Validation, Writing – original draft, Writing – review & editing. LJ: Data curation, Investigation, Supervision, Writing – original draft. FZ: Formal analysis, Investigation, Supervision, Writing – original draft. XH: Formal analysis, Funding acquisition, Investigation, Visualization, Writing – original draft. LZ: Data curation, Investigation, Validation, Writing – original draft. JZ: Methodology, Resources, Writing – original draft. LX: Methodology, Resources, Writing – review & editing, Writing – original draft.
