# Supplementary material for: Tumor growth manifested in two-fifths of low-risk papillary thyroid microcarcinoma patients during active surveillance: data from a tertiary center in China
Source: Front Endocrinol (Lausanne). 2024 Mar 21;15:1359621. doi: 10.3389/fendo.2024.1359621 (PMC10991742; doi:10.3389/fendo.2024.1359621)
Supplement: Supplementary file 2 [file Table_2.docx]

| **Supplementary table2.** Ultrasonographic findings and outcomes of observation in doubling rate for papillary microcarcinoma | | | | |
| --- | --- | --- | --- | --- |
| Ultrasonographic findings | Change in doubling rate of the tumor | | | p value |
|  | A < 0.1 (n=123) | 0.5>A≥0.1 (n=73) | A ≥0.5 (n=23) |  |
| Pattern of calcification |  |  |  |  |
| No calcification | 54 | 34 | 13 |  |
| Microcalcification | 41 | 25 | 6 | NS |
| Macroscopic or rim calcification | 23 | 14 | 4 |  |
| Vascularity |  |  | |  |
| None | 46 | 29 | 5 | NS |
| Inside | 26 | 21 | 8 |  |
| Circumambient | 47 | 18 | 8 |  |
| Both | 2 | 3 | 1 |  |
| Echo |  |  | |  |
| Hypoechoic | 119 | 69 | 20 | NS |
| Iso-echogenicity | 1 | 2 | 1 |  |
| Others | 3 | 0 | 0 |  |
| Margin |  |  | |  |
| Irregular | 62 | 29 | 13 | NS |
| Partial regular | 18 | 10 | 3 |  |
| Regular | 41 | 30 | 7 |  |
| Composition |  |  | |  |
| Solid | 98 | 58 | 21 | NS |
| cystic | 2 | 0 | 0 |  |
| Mix | 2 | 3 | 0 |  |
| Proximal to the posterior capsule |  |  | |  |
| Yes | 33 | 15 | 1 | NS |
| No | 49 | 37 | 12 |  |
| Position of the posterior capsule |  |  | |  |
| Cling to | 24 | 3 | 5 | NS |
| Break off | 2 | 2 | 2 |  |
| Not clinging to | 58 | 46 | 6 |  |
